# Narcissism and consumer behaviour: a review and preliminary findings

**DOI:** 10.3389/fpsyg.2014.00232

**Published:** 2014-03-21

**Authors:** Sylwia Z. Cisek, Constantine Sedikides, Claire M. Hart, Hayward J. Godwin, Valerie Benson, Simon P. Liversedge

**Affiliations:** Centre for Research on Self and Identity, School of Psychology, University of SouthamptonSouthampton, UK

**Keywords:** narcissism, consumerism, eye movement, self-motives, symbolic goods

## Abstract

We review the literature on the relation between narcissism and consumer behavior. Consumer behavior is sometimes guided by self-related motives (e.g., self-enhancement) rather than by rational economic considerations. Narcissism is a case in point. This personality trait reflects a self-centered, self-aggrandizing, dominant, and manipulative orientation. Narcissists are characterized by exhibitionism and vanity, and they see themselves as superior and entitled. To validate their grandiose self-image, narcissists purchase high-prestige products (i.e., luxurious, exclusive, flashy), show greater interest in the symbolic than utilitarian value of products, and distinguish themselves positively from others via their materialistic possessions. Our review lays the foundation for a novel methodological approach in which we explore how narcissism influences eye movement behavior during consumer decision-making. We conclude with a description of our experimental paradigm and report preliminary results. Our findings will provide insight into the mechanisms underlying narcissists’ conspicuous purchases. They will also likely have implications for theories of personality, consumer behavior, marketing, advertising, and visual cognition.

“Some people think luxury is the opposite of poverty. It is not. It is the opposite of vulgarity.”

– Coco Chanel

## INTRODUCTION

The literature indicates that consumer behavior cannot be adequately described by rational models or bounded rationality models (Arnould and Thompson, 2005; Strack et al., 2006). We advocate a complementary approach based on the influence of self-relevant considerations on purchasing decisions. We focus on the construct of narcissism (a self-aggrandising and manipulative interpersonal orientation) and show that narcissism is related to materialism. We further argue that narcissists engage in conspicuous consumption, at least in part, as an attempt to address underlying insecurities. Narcissists’ proclivity for products with symbolic value (e.g., luxurious, flashy) over products with instrumental value (e.g., practical, common-looking) may soothe such insecurities. We describe a novel line of research that addresses these issues and delineate plans to investigate them via eye-tracking methodology. Our plans will allow us to determine whether there are individual differences in the information processing behavior of narcissists relative to non-narcissists when choosing a potential product to purchase.

## CONSUMER DECISION-MAKING AND SELF-RELATED MOTIVES

As long ago as 1890, William James stated that material possessions play an important role in defining the self, and may even become an extension of one’s self. If this is so, we would expect self-relevant considerations (e.g., self-enhancement, self-esteem, self-consistency) to influence the acquisition of material goods. Traditional models of consumer behavior have paid mostly lip service to such motives. These models focused to a large degree on rational economic behavior, where consumers were depicted as engaging in deliberate cost-benefit analysis before deciding to purchase goods (Deaton and Muellbauer, 1980; see also Zinkhan, 1992). Consumers, according to these models, are driven mostly by utilitarian motives. As such, consumers purchase products that deliver what they promise to do (i.e., functionality), are easy to use (i.e., practicality), and are relatively inexpensive (i.e., affordability). In other words, consumers strive for “best value for money.” If the choices they make are not always optimal, bounded rationality theory could come to the rescue. According to this theory, situational constraints (e.g., time pressure, restricted access to information, overabundance of information, and susceptibility to cognitive biases) limit the rationality of consumer decision making (see for example Strack et al., 2006). Nevertheless, from this theoretical standpoint, the decision to buy or not to buy is still considered a consequence of utilitarian concerns or economic considerations, even if such a decision is impaired to some degree by inaccuracy or error.

Traditional models of consumer behavior have been challenged and receded to the background, as they were unable to adequately describe purchasing decisions and were largely unsupported by data. More recent research on consumer behavior has yielded evidence consistent with James’ (1890) original proposals. This research has demonstrated that consumer attitudes towards products and consumer shopping behavior are not only determined by their functional features but also, and perhaps even more so, by their symbolic features (Sirgy, 1982; Belk, 1988; Kressman et al., 2006; Sedikides et al., 2007). People do purchase products for pragmatic reasons, that is, merely as a way to cope more effectively with the necessities of everyday life; however, people also purchase products as means to define and express themselves (Belk, 1985; Dittmar, 1992; Aaker, 1996), to regulate their moods or emotions (Dittmar and Drury, 2000; Dittmar, 2011), to bolster their self-esteem or gain social status (Banerjee and Duflo, 2007; Sedikides et al., 2007), and to fulfil their needs for self-efficacy or mastery (Dittmar, 2011). Thus, self-oriented considerations often underlie consumer decision and behavior.

This self-oriented perspective on consumer behavior is gaining momentum. Dunning (2007a) argued that consumers are not merely rational or “cold” decision-makers, who evaluate and choose consumer alternatives according to standard economic principles. Rather, consumers are motivated agents, driven to make decisions consistent with their “sacrosanct beliefs,” self-images, and self-motives. Consumers make purchases that allow them to view themselves (and be viewed by others) as competent, endearing, and honorable. This notion helps to explain many purchase decisions that would be difficult to comprehend from the point of view of traditional models of consumer behavior. Several theoretical formulations, backed by empirical evidence, concur with such a notion.

Self-congruity theory states that various products and brands are perceived as having certain “personality” traits that in turn reflect the traits of their users. According to this theory, consumers making their buying decisions attempt to choose brands that match their own self-image be it actual or ideal. Self-congruity is guided mostly by two self-related motives: self-consistency and self-esteem (Sirgy, 1982; Malhotra, 1988; Kressman et al., 2006). The self-consistency motive drives individuals toward purchasing products that match the way in which they perceive themselves, that is, products that fit with their preferences, life-styles, and dispositions. Buying products characterized by features that closely match consumers’ desired identities reduces discrepancies between their ideal and actual self and thus boosts their self-esteem.

Complementing self-congruity theory, the purchasing and using of material goods is regarded by symbolic self-completion theory (Wicklund and Gollwitzer, 1982) as a compensation strategy. This theory postulates that, whenever self-discrepancies between the actual and ideal self are detected, or one’s self-image is threatened, compensation motivation becomes activated. This motivation may lead to the acquisition of material possessions, which will either cover (at least momentarily) the detected self-inadequacies or soothe the acute self-threat. For example, people who aspire to master an activity (e.g., playing golf), but are not yet good at it, may purchase expensive equipment and gadgets related to this activity (e.g., golf clubs or clothing) in order to compensate for their undeveloped skills, and thereby promote a more favorable impression of themselves to others and perhaps to themselves.

Symbolic self-completion theory further proposes that individuals are more likely to become materialistic, if their self-concepts are uncertain or threatened (Wicklund and Gollwitzer, 1982). Materialism is defined as “the importance a consumer attaches to worldly possessions” (Belk, 1984, p. 291). The construct has been operationalized as holding the belief that acquiring conspicuous goods is a major route to success, esteem, and happiness (Richins and Dawson, 1992). The implication is that consumers chase prosperity and obtain material goods in an attempt to substantiate their self-definitions. The literature is consistent with this idea. Insecurity experienced as a result of death cognitions (i.e., fear of death; Kasser and Sheldon, 2000) or simply in dreams (Kasser and Kasser, 2001) is positively associated with materialistic attitudes. In addition, mere exposure to self-threatening words (e.g., doubtful, incompetent) activates materialistic attitudes (Chang and Arkin, 2002). Materialism has also been linked with lower self-esteem (Richins and Dawson, 1992; Kasser, 2002), and experimental inductions of low self-esteem or insecurity lead to a materialistic orientation (Braun and Wicklund, 1989; Chaplin and John, 2007). Loneliness also contributes to materialism. In a longitudinal study, loneliness was associated with an increase in materialism over time and, simultaneously, materialism was associated with an increase in loneliness over time (Pieters, 2013). Finally, materialism is associated with negative emotions, such as sadness: individuals who focus on their sadness spend higher amounts of money for the acquisition of material possessions (Cryder et al., 2008).

People may strategically use materialistic possessions to compensate for self-image impairment. In a study by Dong et al. (2013), participants used consumer goods to reinstate their positive identities or at least “hide their faces” so as to minimize negative emotions following embarrassing incidents. Participants were engaged symbolically in these coping tactics by choosing products that either literally covered their faces (e.g., sunglasses) or products that improved and restored their appearance (e.g., cosmetics). Furthermore, the tactic that participants employed had meaningful psychological consequences for their future behavior. Symbolically repairing one’s face by choosing adequate products diminished their aversive emotions (e.g., embarrassment) and restored their willingness to interact with others, whereas symbolically hiding their face had little impact on their emotions and engagement in social behavior. Similarly, Sivanathan and Pettit (2010) demonstrated that individuals resort to high-status and conspicuous goods in an attempt to restore their threatened self-image, thus treating consumption as an indirect source of self-affirmation (Sherman and Cohen, 2006). These studies establish that individuals can strategically use material goods in the service of self-related considerations or motives.

The relation between the self and materialism may have become more prevalent in contemporary life with the rise and dominance of globalization (Dunning, 2007b). Western and Eastern societies alike value money, expensive acquisitions, and attractive features, and consider them valid indicators of success and happiness. Affluent life styles are associated with autonomy, control, high achievement, and desirable states of well-being. Furthermore, various products and services are commonly advertised as necessary bridges toward the positive self and as a *sine qua non* for blissful existence, free from self-worth anxieties and worries (Dittmar, 2011). In all, the influence of self-related motives on marketing decisions is probably on the rise. This said, of course, the theoretical and empirical landscape is bound to be nuanced. For example, the strength of the influence of self-related motives on consumer behavior is moderated by various personality traits, such as level and contingency of self-esteem or self-concept clarity (Dunning, 2007a, b). We propose yet another moderator: narcissism (Sedikides et al., 2007).

## NARCISSISM DEFINED

We view narcissism (subclinical narcissism, to be exact) as a normally distributed personality trait. Narcissism is typically operationalized with the Narcissistic Personality Inventory (NPI; Raskin and Terry, 1988). For convenience, we will refer to persons scoring high on the NPI as narcissists and to those scoring low on the NPI as non-narcissists.

We define narcissism as an agentic, egocentric, self-aggrandizing, dominant, and manipulative orientation (Emmons, 1987; Sedikides et al., 2004). Narcissists have highly inflated and unrealistically positive self-views and feel entitled. They also lack regard for others, showing a diminished interest in affiliation, communal values, and pro-social behavior (Campbell and Foster, 2007; Cisek et al., 2008). Indeed, narcissism is positively associated with antagonism, aggression, and hostility towards others (especially outperforming or critical others), and is negatively associated with agreeableness, empathy, and intimacy (Sedikides et al., 2002; Morf et al., 2011; Hepper et al., in press).

Narcissists are addicted to self-esteem (Baumeister and Vohs, 2001) and to striving towards self-enhancement (Sedikides and Gregg, 2001). They manifest exhibitionism, vanity, and a relentless need to validate their overly favorable self-beliefs in the presence of others (Wallace and Baumeister, 2002). They are also status-oriented and power-driven (Bradlee and Emmons, 1992; Horton and Sedikides, 2009). In order to maintain their excessively positive self-views, narcissists rely on several self-regulatory strategies. They engage in grandiose self-displays (e.g., boasting), flaunt their material possessions, and associate with high-status others (Buss and Chiodo, 1991; Campbell, 1999).

## NARCISSISM, MATERIALISM, AND CONSPICUOUS CONSUMPTION

Narcissists display their affluence as a self-presentational tactic. After all, carefully chosen materialistic possessions can symbolize an individual’s traits, skills, preferences, values, and personal goals, thus differentiating them from others and portraying them as unique and special. Material possessions constitute a rich source of information about others’ identity (Burroughs et al., 1991), and may successfully (and to some degree accurately) express one’s actual and ideal selves. This is so, because material goods convey clues that can be used to make inferences about the owners of these goods. For example, affluent individuals are judged as capable (e.g., intelligent, competent) and sophisticated (e.g., cultured, knowledgeable; Christopher and Schlenker, 2000). Such attributes are highly valued by narcissists, given their agentic inclinations (Sedikides et al., 2002). Affluent individuals are at the same time perceived as relatively inconsiderate (e.g., unkind, unhelpful, unlikable; Christopher and Schlenker, 2000), but this is a price that narcissists are willing to pay given their lack of communal proclivities (Sedikides et al., 2002). Narcissists’ self-worth is contingent on the admiration that they receive from others rather than on building long-lasting relational bonds or on gaining genuine social approval (i.e., respect). Consistent with this reasoning, Lee et al. (2013) demonstrated that narcissists’ consumer decisions are guided by their need to distinguish themselves positively from others. They do so by purchasing goods that are scarce, unique, exclusive, and customizable. They perceive acquisition of such goods as an opportunity to validate, sustain, and elevate their positive self-image.

Taking all this into account, it is perhaps unsurprising that narcissism is associated with materialism (Sedikides et al., 2011) and proneness to compulsive buying (Rose, 2007). Narcissists openly report more interest in pursuing wealth and social status than in pursuing affiliation and communal endeavors (Kasser and Ryan, 1996). They desire material possessions (Cohen and Cohen, 1996) and have high economic aspirations (Roberts and Robins, 2000), prioritizing financial goals, such as attaining a prestigious and well-paid job or securing high standards of living, over social goals, such as helping or teaching others. Similarly to materialistic individuals, they are prone to purchasing high-status and expensive products, which are likely to signal status and sophistication (Richins, 1994). Thus, many features of narcissism mirror materialism, suggesting that both narcissists and materialists engage in conspicuous consumption in an effort to boost their status, self-protect, or derive self-esteem from the responses of admiring others.

We have reviewed literature showing that narcissism is linked with consumer behavior. Conversely, it has been suggested that the emphasis on consumerism prevalent in contemporary society sparks increases in narcissism. Lasch (1991) labeled contemporary society as the “culture of narcissism.” He argued that the culture is characterized by an “entitlement mentality” that comprises an unjustified sense of privilege. This, in turn, pushes consumers further into a vicious circle of excessive consumption that feeds their self-images. Indeed, there is evidence that levels of narcissism are on the rise both in Western culture (Twenge et al., 2008) and in Eastern (i.e., Chinese) culture (Cai et al., 2012).

As narcissists are predominantly concerned with agentic (rather than communal) traits and goals, their materialism may further strengthen this orientation. Focusing on money enhances individuals’ self-sufficiency, but diminishes their communal motives and pro-sociality. In particular, participants primed with money (vs. those in a control condition) are less likely to ask for help and to help others, prefer to work and play alone, and keep a larger physical distance between themselves and others (Vohs et al., 2006). Also, cues that prompt materialism inhibit social values and increase competitiveness (Bauer et al., 2012). Moreover, individuals preoccupied with money are egocentric (Belk, 1985) and often feel alienated and disconnected from others (Kasser, 2002; Pieters, 2013). In summary, materialism may exacerbate the signature narcissistic characteristics: self-sufficiency or autonomy, egocentricity, competitiveness, unwillingness to help, and poor interpersonal relationships.

## COMPENSATING FOR EGO FRAGILITY

One reason why individuals embrace materialism and engage in consumption of prestigious brands may have to do with their attempts to override their inadequacy and to compensate for doubt surrounding their self-worth (Wicklund and Gollwitzer, 1982; Chang and Arkin, 2002). Narcissists routinely present themselves as successful and confident, if not arrogant. However, this direct and bold approach may be partially a disguise for their brittle, fragile selves (Gregg and Sedikides, 2010; Cheng et al., 2013). This inner fragility is often attributed to inadequate parental practices such as excessive adoration or copious neglect (Kernberg, 1975; Kohut, 1976; Horton et al., 2006; Otway and Vignoles, 2006).

There is empirical support for these theoretical proposals. First, narcissists’ affect and level of self-esteem fluctuate more than those of non-narcissists (Bogart et al., 2004; Zuckerman and O’Loughlin, 2009; Zeigler-Hill et al., 2010). Secondly, narcissists, in comparison to non-narcissists, have lower implicit self-esteem (Jordan et al., 2003; Zeigler-Hill, 2006; Gregg and Sedikides, 2010). Thus, narcissist consumer behavior may be seen as a coping strategy to compensate for their self-doubts and insecurities. Through their consumer behavior, they project the positive attributes of their purchases onto their private and public self-image. In particular, they show a distinct consumer behavior pattern, where they accumulate and display material possessions (e.g., flashy, highly fashionable clothes, top-range cars, expensive watches) that bear prestigious labels (Sedikides et al., 2011). On the basis of flimsy evidence, then, we would surmise that Coco Chanel was endorsing a narcissistic consumer behavior pattern in our opening quote.

If the main force guiding narcissists’ conspicuous consumption is rooted in their self-related motives, and specifically serves to fulfill their self-presentational goals, narcissists should be focused on symbolic rather than on instrumental aspects of acquired or potential purchases. Sedikides et al. (2007) hypothesized that narcissists should demonstrate this preference in their consumer choices by selecting ostentatious, fashionable, and flashy versions of a given product over affordable, practical, and modest-looking versions. To test this hypothesis, Cisek et al. (2011) conducted a vignette study in which participants were presented with pictorial and descriptive examples of two alternatives for seven different products (e.g., mobile phones, MP3 players, laptops). One example represented a symbolic choice reflecting superior attractiveness but inferior practicality. The other example represented an instrumental choice reflecting superior practicality but inferior attractiveness. Participants were instructed to indicate their consumer choice for each of these products. The obtained results confirmed that narcissism significantly and positively predicted the number of symbolic products chosen. Thus, narcissists were more concerned with the products’ expressive properties rather than their practical attributes. They selected new and impressive-looking items over reliable and practical ones. Additionally, mediational analyses revealed that this orientation toward symbolic products was accounted for by narcissists’ materialism and self-esteem.

However, although these findings contribute to the emerging literature, a number of important questions remain unanswered. For example, do narcissists employ deliberate thinking in choosing symbolic products in spite of their practical shortcomings? That is, do narcissists make well-informed decisions, consciously processing all available information and sacrificing instrumentalism for the sake of the expressive properties of their choices? Alternatively, do narcissists make rapid decisions, choosing a product that they believe is better on most of the dimensions? This second possibility could be linked to inadequate information processing and could be reflected in an over-reliance upon pictorial clues, which in turn could be related to narcissists’ high levels of impulsivity. It has been documented that impulsive buying is related to materialism (Yurchisin and Johnson, 2004). Evidence also points to a positive relation between narcissism and impulsivity (Vazire and Funder, 2006), although in our own research impulsivity was not found to mediate the link between narcissism and preference for symbolic products (Cisek et al., 2011).

To shed additional light on how information is processed during consumer decision-making, we will measure the amount of time that narcissists and non-narcissists spend looking at the symbolic and instrumental pictures and at written descriptions of potential purchases. We will use objective measures of attention, that is, eye movement data collected via an eye tracker whilst participants complete the task.

## EYE MOVEMENT RESEARCH IN ADVERTISEMENT AND CONSUMER BEHAVIOR

Eye tracking methodology provides an objective record of participants’ eye movements as information is processed on-line. Eye movements are one of the most frequent of all human movements (Bridgeman, 1992): on average, individuals make 3–4 eye movements, known as saccades, per second. These movements are essential to relocate the high acuity area of the retina, the fovea, such that light from new locations in the visual field falls upon it and new objects or areas of objects can be inspected in detail. Hence, eye movements are necessary for efficient operation of the visual system, and there is a close relation between eye movements and ongoing information processing. Eye movement behavior has also been utilized to explore visual cognition in a wide variety of tasks and environments, including mental imagery, memory, language comprehension, reading, reasoning and decision making (Rayner, 1998, 2009a,b; Liversedge and Findlay, 2000). Eye movements can also provide insight into psychiatry, neuroscience, ergonomics, and marketing. In recent years, the increased sophistication and accessibility of eye tracking technologies have led to a substantial number and broad variety of studies that employ eye tracking methods. Applied research has included package design, website design, advertising, computer game design, automotive engineering, evaluating skills of radar operators, pilots, security officers at airports, and many others (Richardson and Spivey, 2004). There is a growing body of research using eye tracking methods to analyze marketing decisions and advertisement perception (Wedel and Pieters, 2000; Rayner et al., 2001, 2008; Radach et al., 2003).

Eye movements are driven both by the characteristics of the task, and the properties of visual stimuli. For example, a classic study by Yarbus (1967) showed that the nature of the task influences the individuals’ eye movements such that different patterns of saccades and fixations (where the eyes remain stable to process information) are observed when the same picture or scene is viewed under different inspection instructions (see also Hayhoe and Ballard, 2005; Underwood and Foulsham, 2006). Also, participants’ fixations center on informative or interesting areas of the image, whereas blank or uniform regions are often left uninspected (Wooding, 2002; Richardson and Spivey, 2004). At the same time perception is influenced by memories, beliefs, attitudes, emotions, expectations, and other forms of top-down knowledge that viewers “bring” to the given image. However, advertisement research has addressed not only the visual patterns of examining pictorial information, but also reading text that often accompanies and forms an important part of such adverts. How long an observer spends initially fixating an area or object (gaze duration), how quickly a saccade is initiated to a stimulus (saccade latency), and the occurrence of revisits or regressions (saccades that return to previously fixated areas) can be analyzed to provide understanding of the online cognitive processing of pictorial information and text reading (Just and Carpenter, 1980; see also Rayner, 1998). In summary, eye movement tracking can provide data that will help to identify factors that determine the amount of attention allocated to different types and elements of adverts. This is critical especially in light of research showing that gaze durations to different advertisements correlate positively with subsequent product choices (Treistman and Gregg, 1979; Lohse, 1997). Put simply, when choosing to purchase a product, total fixation time increases for that product.

Surprisingly, although there is a substantial body of research on eye movements during reading processes and during examination of a scene or an image, there are relatively little data referring to integration of text and pictorial information, and these data are not always conclusive or free from ambiguity. Rayner et al. (2001) showed that advertisement viewers very quickly fixate the text of an advertisement. They also spend more time reading the text of the advertisement than inspecting pictorial information, especially if the ads are relevant to their goals (e.g., buying a given product). However, Radach et al. (2003) reported different findings. They observed that participants made repeated fixations back and forth between the different elements of an advertisement (that is headline, text, and picture) during inspection. More recently, and in contrast to the study from 2001, Rayner et al. (2008) demonstrated that participants who were asked to assess the quality of advertisements spent more time analyzing pictorial parts of the adverts than the accompanying text, and fixated the images first within a trial, and only after a relatively long time inspecting these did they move their eyes to the linguistic information provided. This difference in visual examination patterns has been accounted for by differences in the nature of the task (that is, the active goal of the viewer) and the type of the advertisements. Rayner et al. (2008) argued that participants in the first study were instructed to make a consumer decision, that is to decide whether to buy a particular product, and in that case they paid more attention to linguistic than pictorial descriptions of the products. Participants in the two other studies (Radach et al., 2003; Rayner et al., 2008) were asked to assess the attractiveness or effectiveness of advertisements, and here they generally showed an inverse pattern of visual investigation. However, although the given instructions influenced how long participants fixated different aspects of the advertisements, the advertisements themselves also influenced the patterns of eye movements. These results render conclusions less firm and in need of further investigation.

## PROPOSED EYE MOVEMENT STUDY TO EXAMINE NARCISSISTIC CONSUMER CHOICES

To understand better information processing and the influence of self-related motives on consumer decisions, we will examine the amount of time that narcissists and non-narcissists spend looking at various forms of information related to their potential purchases. Our study aims to investigate consumer choices made by narcissists and non-narcissists and whether possible differences in such choices are accompanied by differences in online cognitive processing of the information, as indexed by eye movement patterns. Our primary goal is to find out whether narcissists (in contrast to non-narcissists) spend more time fixating pictorial rather than linguistic information about available product alternatives, and whether they spend more time fixating symbolic rather than utilitarian products. Participants in this study will be asked to make consumer decisions as if they intended to buy given products.

Participants will be undergraduate students of the University of Southampton. At the beginning of each academic year, Psychology undergraduate students complete a range of personality questionnaires, including measures of narcissism (NPI; Raskin and Terry, 1988) as well as self-esteem (Rosenberg Self-Esteem Scale; Rosenberg, 1965), and materialism (Richins and Dawson, 1992).

Participants scoring either in the top third or bottom third of the NPI will be invited to take part in the study. Firstly, they will be requested to complete several extra personality scales, such as the Pathological Narcissism Inventory (Pincus et al., 2009), and Hedonistic Consumption Scale and Impulsive Trait Scale (Hausman, 2000). This will allow us to control for extraneous variables when analyzing narcissistic patterns of consumer behavior.

Subsequently, participants will take part in an eye movement experiment. A computer screen will display the stimulus material. On each trial, two examples of a given product will be presented; one symbolic version and one instrumental version of the same product (with each product featuring a pictorial and linguistic description). By “symbolic,” we mean visually attractive, flashy, and fashionable items with inferior practicality. Instrumental products will be described as practical and economical, but will be much less visually appealing. (The differences in perceptions of visual attractiveness as well as practicality and usefulness of the two types of products have been established in extensive pilot-testing). There will be seventeen trials in total, each depicting a different product, such as a sofa, toaster, laptop, watch, coffee machine. Three of the seventeen products (digital book reader, roller-case, and cross-trainer) will have one alternative that is superior on both dimensions: attractiveness and practicality. These products will be introduced to disturb the pattern of one product always being less attractive but more practical than the other one, and to minimize the chance of participants guessing the purpose of the experiment. The arrangement of the examples on the trial display screen will vary such that the pictures will either appear above or below the written descriptions, and the symbolic and instrumental products will appear either on the left or the right of the screen (counterbalanced). The order of products will be randomized. Participants will be instructed to look at the pictures, read the descriptions carefully, and choose which product they would most likely buy. The eye tracker will record every eye movement and fixation made during inspection for each trial. Analyses of the eye movements will allow us to determine which elements (picture/description of symbolic/ instrumental product) are fixated initially, providing an index of which type of element is of the highest relevance to the participants completing the task. Furthermore, the eye movement analyses will also enable us to explore the amount of time spent fixating, the number of fixations, and the fixation durations upon the different elements within the display, thereby allowing us to investigate the depth, extent, and time course of information processing on the different elements as a function of narcissism. We will use regression analyses of different behavioral and eye movement measures in order to test whether narcissism predicts a stronger preference for symbolic products.

We hypothesize that narcissists will demonstrate a predilection for symbolic products: they will make more symbolic choices than non-narcissists. Given the relevance of symbolic products for narcissistic self-esteem, we also hypothesize that narcissists will fixate such products for longer, and that generally they will fixate images for longer than the text. Given that participants will be instructed to make their consumer decisions as if they were actually to buy the products, in line with results of Rayner et al. (2001), we expect that non-narcissists will spend longer fixating text than pictorial information.

### PILOT STUDY

We conducted a short pilot study following the procedure described above but without measuring any individual differences, as the goal of this study was to validate the paradigm for use with narcissists at a future date. Seven individuals (students or visitors from the University of Southampton, six females and one male) took part as volunteers without compensation. Due to an intermittent hardware error, the experiment terminated before all trials had been completed for two participants, and it “looped” resulting in additional trials for one additional participant. Given that this was a pilot study, and that these participants’ data patterned in a similar manner to the data of the remaining participants, we included data from all seven participants in the analyses we report below.

Analyses included total fixation time – separately for utilitarian and symbolic alternative – number and average duration of fixations, and probability of a first fixation being in a given region.

The pattern of results replicates that obtained by Rayner et al. (2001) (see **Table 1**). A 2 (type of information: text vs. picture) × 2 (type of product: symbolic vs. utilitarian) repeated-measures ANOVA revealed a statistically significant effect of type of information for the total fixation time, *F*(1,6) = 13.44, *p* = 0.01, $\eta_{p}^{2}$ = 0.69. Participants spent more time fixating on the text (*M* = 5258.24, SE = 1210.91) than on the pictorial representation of products (*M* = 1109.07, SE = 238.40). The main effect of type of product and the interaction were not significant, *F*(1,6) = 2.61 *p* = 0.16, and *F*(1,6) = 0.89, *p* = 0.38, respectively.

**Table 1:** Means and standard deviations (in brackets) for general eye movement parameters as a function of stimulus type in the pilot study.

	Symbolic picture	Utilitarian picture	Symbolic description	Utilitarian description
Mean total fixation time (ms)	1228.33 (732.31)	989.81 (539.84)	5252.97 (3203.77)	5263.51 (3348.91)
Mean number of fixations	5.02 (2.32)	4.26 (1.78)	26.76 (15.23)	26.33 (14.89)
Mean fixation duration (ms)	239.57 (49.19)	226.35 (45.68)	198.70 (27.31)	198.98 (30.40)
Mean percentage of first fixations	31 (0.18)	32 (0.19)	22 (0.21)	13 (0.11)

*We first computed the mean fixation duration for each trial, and then the mean across trials. For this reason, the mean fixation duration we report is not simply the mean total fixation time divided by the mean number of fixations. This approach ensures that the mean value for each trial contributes equivalently to the overall mean value.*

In addition, and consistent with the above result patterns, we obtained a significant main effect of type of information for the number of fixations, *F*(1,6) = 16.15, *p* = 0.007, $\eta_{p}^{2}$ = 0.73. Participants fixated on the text more often (*M* = 26.54, SE = 5.68) than on the pictures (*M* = 4.64, SE = 0.76). Again, the main effect of type of product and the interaction were not significant, *F*(1,6) = 3.25 *p* = 0.12 and *F*(1,6) = 0.12, *p* = 0.74, respectively.

The instruction delivered to participants was very similar to that given to participants in the study by Rayner et al. (2001), and this instructional similarity for the current pilot study may account for the replication. Participants who took the perspective of a potential buyer tried to gain useful information about the characteristics of different products, and thus, focused their attention on the linguistic description of the products to a greater extent than on their pictorial features. As images convey mostly evidence of symbolic values, whilst text provides additional information about practical aspects of a given product, this asymmetry in visual investigation may also suggest that people in general seek information about utilitarian properties when considering buying a product. Furthermore, before making a consumer decision, participants investigated both alternatives of a given product (symbolic and utilitarian) to the same extent. Such a pattern of behavior may be useful in situations when a potential buyer has to decide which product to purchase and has no initial knowledge about available alternatives. After all, to determine if products are or are not practical and useful, a consumer must first collect and process information about these products.

At the same time, participants made significantly longer fixations on pictures (*M* = 232.96, SE = 17.46) than on text (*M *= 198.84, SE = 10.87), type of information *F*(1,6) = 14.61, *p* = 0.009, $\eta_{p}^{2}$ = 0.71. This finding is typical and has also been reported in previous studies (Rayner et al., 2001, 2008). The type of product and the interaction remained non-significant, *F*(1,6) = 2.23 *p* = 0.19, and *F*(1,6) = 2.65, *p* = 0.16, respectively.

We obtained no significant effects for type of information, type of product, or the interaction on the probability of first fixating a region (*F*s < 1.5, *p*s > 0.26). Thus, participants first fixated on each of the different areas of the stimuli with equal probability.

Overall, the results of the pilot study replicate the earlier findings of Rayner et al. (2001), reinforcing the idea that task instruction can drive inspection behavior for product advertisements. The pilot data have served to validate our paradigm for future investigation on how such inspection patterns are modulated by high levels of narcissism. Based on the current data, our paradigm allows us to index online decision-making behavior, and provides an insight into the interplay between human visual sampling behavior and the moment-to-moment cognitive processes in which individuals engage when they make consumer decisions. Specifically, we can evaluate the relative importance of pictorial versus linguistic information when such decisions are made and record which aspects of available information are salient in capturing and holding a consumer’s attention. We hope that our future studies will provide a clear demonstration of the value of eye movement technology in the investigation of the influence of personality traits (i.e., narcissism) on consumer behavior.

## Conflict of Interest Statement

The authors declare that the research was conducted in the absence of any commercial or financial relationships that could be construed as a potential conflict of interest.
